# Copper-Chelated Chitosan Microgels for the Selective Enrichment of Small Cationic Peptides

**DOI:** 10.3390/gels10050289

**Published:** 2024-04-24

**Authors:** Jean-Christophe Jacquier, Ciara Duffy, Michael O’Sullivan, Eugène Dillon

**Affiliations:** 1 UCD Institute of Food and Health, School of Agriculture and Food Science, University College Dublin, D04 V1W8 Dublin, Ireland; 2 Conway Institute of Biomolecular and Biomedical Research, University College Dublin, D04 V1W8 Dublin, Ireland

**Keywords:** copper chelate, chitosan, IMAC, amino acid, cell penetrating peptides, antimicrobial peptides

## Abstract

Copper-chelated chitosan microgels were investigated as an immobilized metal affinity chromatography (IMAC) phase for peptide separation. The copper-crosslinked chitosan beads were shown to strongly interact with a range of amino acids, in a wide range of pH and saline conditions. The beads exhibited an affinity that seemed to depend on the isoelectric point of the amino acid, with the extent of uptake increasing with decreasing isoelectric point. This selective interaction with anionic amino acids resulted in a significant relative enrichment of the supernatant solution in cationic amino acids. The beads were then studied as a novel fractionation system for complex milk hydrolysates. The copper chitosan beads selectively removed larger peptides from the hydrolysate aqueous solution, yielding a solution relatively enriched in medium and smaller peptides, which was characterized both quantitatively and qualitatively by size exclusion chromatography (SEC). Liquid chromatography–mass spectrometry (LCMS) work provided comprehensive data on a peptide sequence level and showed that a depletion of the anionic peptides by the beads resulted in a relative enrichment of the cationic peptides in the supernatant solution. It could be concluded that after fractionation a dramatic relative enrichment in respect to small- and medium-sized cationic peptides in the solution, characteristics that have been linked to bioactivities, such as anti-microbial and cell-penetrating properties. The results demonstrate the use of the chitosan copper gel bead system in lab scale fractionation of complex hydrolysate mixtures, with the potential to enhance milk hydrolysate bioactivity.

## 1. Introduction

There is a huge interest in peptide and protein separation across a wide range of disciplines including pharmaceutical, biomedical and food industries [1,2,3,4]. For example, in the food industry one research area gaining a lot of interest is the separation of bioactive peptides from complex heterogeneous mixtures, such as hydrolysates [5,6]. Typically, techniques are sought that are capable of extracting out the peptides and proteins of value, such as bioactive peptides, from the undesired compounds in the mixtures.

Peptide and protein separation systems used in the food industry to separate out peptides of interest are classically based on physical properties, such as size using nanofiltration (NF) and ultrafiltration (UF) [7,8], or hydrophobicity using solid phase extraction (SPE) [9]. Alternatively, in biomedical sciences, the use of immobilized metal affinity chromatography (IMAC) technique for specific peptide and protein separation has found widespread use and success [10,11,12]. The IMAC technique relies on chemical interactions between the amino acid side chains of peptides or protein molecules and transition metals, such as copper, present in the IMAC system. Copper-based IMAC systems have been prepared in different forms, such as gels and resins [13,14], with the copper loaded onto the system via a chelating or linkage agent [11]. In a comprehensive review by Ueda et al. [11], the authors have made a connection between the number of coordination sites of copper available for interaction and the selective affinity of the system. Typically, the copper is loaded in a way that yields systems with specificity for histidine and histidine containing peptides, which is useful for immunoglobulin recovery in biomedical applications. However, other varying affinities have previously been reported, such as by Muzzarelli et al. [15], in which the IMAC system prepared exhibited a marked selectivity for the anionic amino acids aspartic and glutamic acids, in addition to tryptophan, relative to histidine and other amino acids.

Chitosan is a carbohydrate polymer obtained from the deacetylation of chitin under alkaline conditions, resulting in a structure of a glucosamine backbone with three functional groups: amino groups and primary and secondary hydroxyl groups [16]. It has been established that the active primary amino groups of chitosan have a high binding capacity for transition metals, such as copper [17]. Chitosan has been previously incorporated in IMAC systems, typically as a coating polymer onto silica beads to provide a chelating agent for copper, and which resulted in complicated but useful multi-layer systems [14,18,19]. Alternatively, Bai et al. [20] prepared a promising IMAC system based on a chitosan matrix, in which preformed chitosan gel beads were loaded with copper, and then chemically crosslinked with glutaraldehyde. These chitosan copper beads exhibited a selective affinity for histidine over tryptophan when compared to control chitosan beads without copper, which was attributed to the copper contained in the beads. In contrast to the multi-step manufacture process used for the previous system, a simple method to prepare chitosan copper gel beads was developed recently [21], in which chitosan was crosslinked by chelation to copper sulphate solution to form gel beads directly. The copper crosslinked chitosan beads had high levels of copper bound (as high as 95 mg/g bead), which was found to be strongly chelated within the chitosan matrix when tested in a range of saline aqueous media, and at different pHs. Nevertheless, the possibility of using these copper crosslinked chitosan beads to selectively separate amino acids and peptides based on chemical interactions has not yet been explored. 

Therefore, the objective of the current study was to determine if copper crosslinked chitosan gel beads would be suitable for use in peptide and protein separation, by determining first if the beads have specific interactions with amino acids. The nature of these interactions was further investigated by examining the parameters affecting uptake, such as concentration, pH and salt. This also served to evaluate the feasibility of the beads to operate under conditions typical of unpurified mixtures found in food applications. Finally, the competitive immobilization of peptides from a complex mixture such as hydrolysate was investigated to evaluate the potential of these beads as a selective affinity separation technique.

## 2. Results and Discussion

### 2.1. Amino Acids Immobilzsation

**Figure 1:**
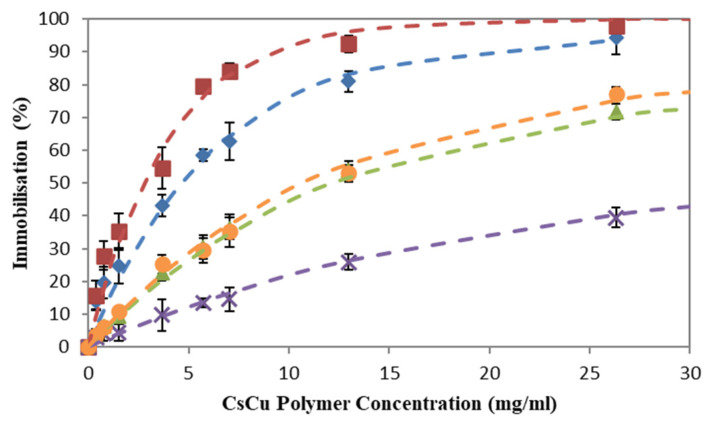
Immobilization of (♦) Trp, (■) Asp, (▲) His, (**X**) Lys and (●) Ala in the gel beads with increasing chitosan copper polymer concentration (mg/mL). Dashed lines are visual fits only.

The chitosan copper beads could successfully immobilize the range of amino acids at all chitosan copper polymer concentrations, and for all amino acids the extent of immobilization increased with increasing chitosan copper polymer concentration (Figure 1).

However, different immobilization profiles can be observed for the five amino acids investigated in the current study. There is relatively higher immobilization of aspartic acid and tryptophan at lower chitosan copper polymer concentrations compared to the other amino acids, as the beads immobilized over 80% of the available amino acid at a chitosan copper polymer concentration of 13 mg/mL. Of the five amino acids used, lysine is observed to have the lowest specific affinity for the chitosan copper bead matrix, as at the maximum chitosan copper polymer concentration (26 mg/mL) used, only 39 ± 3% of the total lysine was immobilized into the bead. In the current study, the beads exhibited the highest affinity for aspartic acid, as the amino acid was immobilized to a much higher extent than histidine or lysine. These results are alternative to what is typically reported in the literature for IMAC techniques involving copper, where a relatively high affinity to histidine (and histidine containing peptides/proteins) relative to other amino acids has been reported, as detailed in a review by Ueda et al. [11]. The current results show a different affinity, which could potentially have been influenced by the way the copper is chelated to the chitosan bead matrix. It could be that the chitosan matrix is crosslinked via copper chelation in such a way that the available coordination sites of copper are reduced, as interpreted through the relatively lower affinity for histidine (Figure 1). It has been reported previously by Ueda et al. [11] that a reduced number of coordination sites available translate to a reduced recovery of histidine. Interestingly, the affinity of the current system seems to follow a trend, in which the extent of immobilization increases with decreasing isoelectric point of the amino acid.

#### 2.1.1. Effects of pH on Amino Acid Immobilization

**Figure 2:**
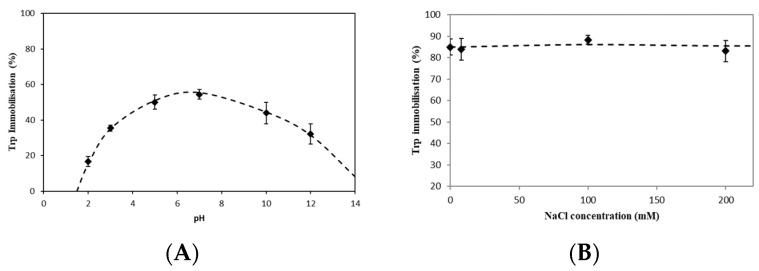
Effects of pH (**A**) and neutral salt (**B**) on the immobilization of tryptophan in chitosan copper bead. Dashed lines are visual fits only.

The effect of pH on the immobilization of tryptophan was investigated in order to see if the charge on the amino acid could influence its immobilization. Figure 2A shows the immobilization of tryptophan in beads with increasing pH from 2 to 12. Tryptophan was successfully immobilized into the chitosan copper beads over a wide pH range investigated, although the extent of immobilization varied. The highest immobilization was found to occur over the pH region of pH 5–10, which is also the pH range where tryptophan is overall neutral. There was a decrease in the immobilization of tryptophan at each of the most extreme pH values investigated, pH 2 and pH 12. A relatively lower immobilization rate was measured at the most acidic pH 2, which may be due to copper ions beginning to be released from the chitosan matrix into the surrounding solution [21]. At the most alkaline condition there was also a relative decrease in immobilization, but not to the same extent as at the acidic pHs. A possible reason may be the slight precipitation of copper from the matrix as copper hydroxides at these high pH values.

#### 2.1.2. Effects of Salt on Amino Acid Immobilization

The effect of salt on the immobilization of tryptophan was investigated in order to confirm if electrostatic interactions are involved and thus provide further insight on the immobilization mechanism. Figure 2B shows the immobilization of tryptophan with increasing salt (NaCl) concentration, while keeping both chitosan (4 mg/mL dry basis) and tryptophan (100 mg/L) concentrations constant. The immobilization at this polymer concentration is observed to be independent of salt concentration and overall remains constant, with only minor fluctuations over the range of salt concentration studied.

From the current results it can be postulated the salts did not compete with tryptophan, indicating that the primary interaction is not electrostatic in nature. If electrostatic interactions were involved, the presence of competing ions would displace the amino acids from the beads, as had been observed for the encapsulation of histidine by whey protein microbeads [22]. In contrast, O’ Neill et al. [23] reported an increased uptake of the bioactive riboflavin into whey protein microgel beads in the presence of a salt (calcium chloride). The uptake of riboflavin was, therefore, suggested to be due to hydrophobic interactions as when the surrounding solution increased in polarity due to the presence of salts, the riboflavin was driven into the microgel beads. The results of the current experiment would indicate that the immobilization of tryptophan into the copper-crosslinked chitosan beads is neither due to electrostatic nor hydrophobic interactions. 

The ability of the beads to operate under saline conditions is highly advantageous for applications in both the food and biomedical disciplines, where working material may not be pure, increasing cost efficiency as solutions are not required to be desalted prior to immobilization techniques.

In the current experiments, the post-contact solution has been relatively enriched in alanine, histidine and lysine relative to aspartic acid and tryptophan, which are relatively more concentrated in the bead, i.e., there has been a dual concentration effect. In the pre-contact solution, all the amino acids were present at equal ratio of 1:1, whereas, for example, post-contact with the beads the lysine: aspartic ratio of the solution has been increased 2.2:1. The post-contact depletion of anionic amino acids results in a relative enrichment of the cationic amino acids. Interestingly, some structural properties reported for bioactive peptides have included hydrophobic amino acid residues, in addition to lysine groups [24]. More specifically, relatively small cationic peptides rich in lysine, arginine and histidine have been reported to possess antimicrobial properties [25,26]. The opportunity to enrich the bioactive peptides in the post-contact solution reduces the processing steps compared to classic IMAC separation techniques, as the compounds of interest do not need to be subsequently extracted from the beads. The selective affinity displayed in the current study presents an alternative IMAC system for the immobilization of specific amino acids not as widely reported, which could be hugely useful in the fractionation of complex protein mixtures, or hydrolysate solutions containing many bioactive peptides.

### 2.2. Hydrolysate Immobilzsation

**Figure 3:**
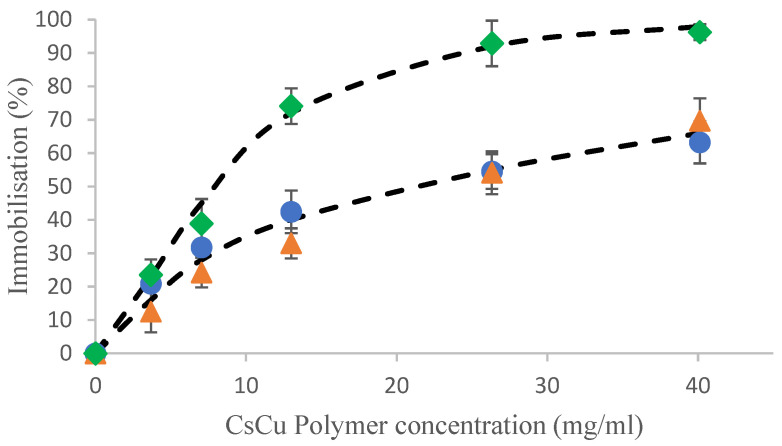
Immobilization in chitosan copper polymer beads of sodium caseinate (♦), and a casein hydrolysate measured either by UV absorption (▲) or by SEC with OPA derivatization (●). Dashed lines are visual fits only.

While we have shown that the chitosan copper beads were capable of strongly interacting with a range of simple amino acids, the results in Figure 3, importantly, show that the beads are also capable of interacting with larger, intact proteins, such as caseins. Further, the extent of uptake in the current experiment is also very comparable to the uptake reported for the amino acids. However, the physical location at which the interactions between the protein and bead are occurring will most likely be different to that of the amino acids, as larger proteins are not known to diffuse into gels. Due to these physical constraints, the uptake of the proteins will most likely be via interactions with, or adsorption onto, the surface of the bead matrix.

Similarly, the casein hydrolysate was also shown to interact significantly with the chitosan beads, although to a lesser extent than the intact protein (Figure 3). In order to qualitatively assess if components of the hydrolysate are being selectively taken up, size exclusion chromatography (SEC) was performed to analyze the hydrolysate solutions pre- and post-contact with the beads and to give a molecular weight profile of the hydrolysate and deduce if the beads have a selective affinity for certain peptides. However, for the chromatographic results to be representative, the overall uptake by chromatography needed to be validated by comparison to the quantitative measurements previously done by the UV method. Figure 3 shows the uptake as quantified by the SEC compared to the uptake as quantified by the UV method and it can be observed that there is excellent agreement between methods and that both are equally valid methods for quantitative assessment of uptake. 

#### 2.2.1. Characterization of the Immobilized Peptides Molecular Weights by SEC

**Figure 4:**
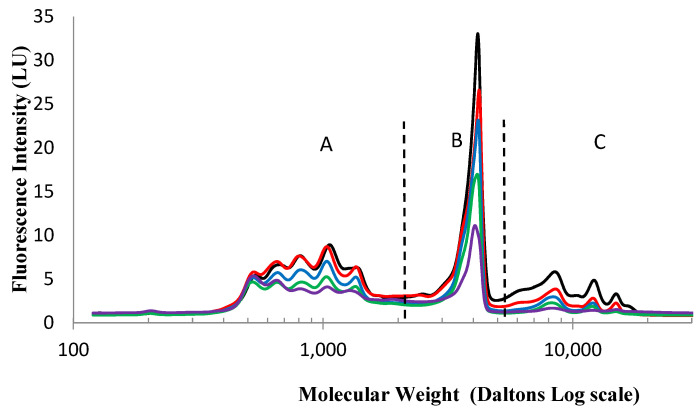
Size exclusion chromatography profiles of the precontact sodium caseinate hydrolysate (**—**) and the post-contact solutions at a range of chitosan copper polymer concentrations (**—**) 4 mg/mL, (**—**) 13 mg/mL, (**—**) 26 mg/mL, (**—**) 40 mg/mL. The profile is divided into zones; A ≤ 2000 Da, 2000 Da ≤ B ≤ 5000 Da and C ≥ 5000 Da.

Figure 4 shows the molecular weight distribution of the original hydrolysate solution in addition to the post-contact or supernatant solutions containing the non-immobilized peptides at a range of increasing chitosan copper polymer concentrations from 0 to 40 mg/mL.

It is evident from Figure 4 that there are three distinct zones in the size distribution profile of this particular sodium caseinate hydrolysate, Zone A with small peptides ≤ 2000 Da, Zone B with intermediate sized peptides between 2000 and 5000 Da and Zone C containing large peptides over 5000 Da. Also, when comparing the peptide profiles of the solution post-contact, there seems to be a relatively higher uptake of the larger peptides (zone C), when compared to the extent of uptake of the medium and smaller peptides and amino acids (zone A and B). This can be observed visually from the relatively steeper decrease in the peak area of zone C going from the pre-contact to the post-contact solution, compared to the decrease for the other zones, indicating that the majority of the smaller and medium sized peptides in the solution have not interacted with or been immobilized by the beads. Therefore, the beads can be said to exhibit a selective affinity for the larger peptides in the hydrolysate (Zone C in Figure 4). 

**Figure 5:**
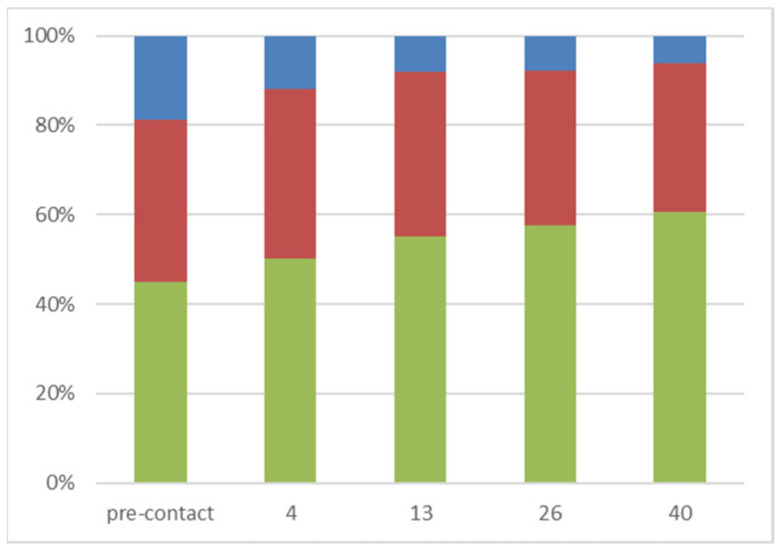
Molecular weight distribution of the precontact sodium caseinate hydrolysate and the post-contact solutions at a range of chitosan copper polymer concentrations 4 mg/mL, 13 mg/mL, 26 mg/mL and 40 mg/mL. The profile is divided into zones; A ≤ 2000 Da (green), 2000 Da ≤ B ≤ 5000 Da (red) and C ≥ 5000 Da (blue).

This pattern of selectivity is more easily visualized when a graph of the proportion of the three peptides size categories in the solutions post-contact is represented with increasing chitosan concentration (Figure 5). This figure shows clearly that the large peptide fraction is selectively more immobilized by the chitosan copper beads, thereby showing a steep decrease in the post-contact solution, contrary to the small peptide fraction which is comparatively enriched in the supernatant solution.

This pattern of selectivity is slightly difficult to explain, as it might be expected that the smaller peptides would have a better opportunity to permeate the entirety of the bead and, therefore, would show a higher depletion in the post-contact solution. Instead, the reverse is observed. One possible reason for the increased interaction between the beads and the larger peptides could be because of multiple binding sites on the large peptides leading to cooperative binding onto the beads. At the chitosan copper polymer concentration of 4 mg/mL, there is the relatively largest proportional difference in the size profile. This leaves a post-contact hydrolysate solution where there has been a large proportional increase, or relative enrichment, in the smaller peptides and amino acids.

#### 2.2.2. Characterization of the Enriched Peptides by LCMS

To fully evaluate the use of the chitosan copper beads as a fractionation method to enrich bioactive peptides, the characteristics of the peptides in the post-contact solutions must be studied in more detail. As the large peptides have been removed by the beads with the smaller peptides relatively enriched in the post-contact solution, it is desirable to characterize the smaller fraction to determine if the peptides possess any attributes that are linked to bioactivity. To elucidate the characteristics of the peptides in the post-contact solution, such as amino acid sequences and charge states, LCMS was employed. Specifically, electron spray ionization mass spectrometry (ESIMS) HPLC was selected to characterize the pre- and post-contact solutions, as this technique is more suited to the mass ranges of interest, compared to other methods like Maldi TOF.

**Figure 6:**
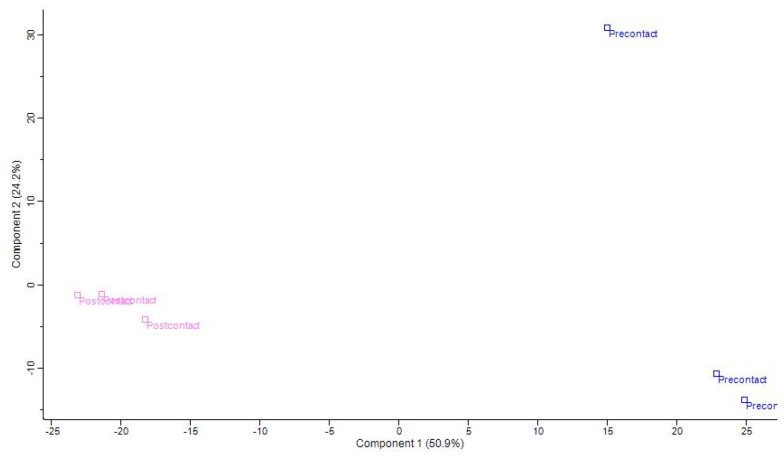
Principal component plot (PCA) of the triplicate pre-contact (blue) and post-contact (pink) solutions after being fractionated at a chitosan copper polymer concentration of 4 mg/mL.

Overall, 185,030 peptide-like features (PLFs) for post-contact samples and 171,465 PLFs for pre-contact were observed throughout the entire mass spectrometry run. From these 356,495 PLFs, 44,296 peptides with a charge state greater than one were sequenced, representing 12.68%. After matching to the database with an FDR of 0.01, 1057 peptides were identified across the experiment. Principal component analysis (PCA) (Figure 6) was employed for visualization of global peptide signatures across the two groups and to examine systematic trends with the data. The principal two components identified accounted for 50.9% and 24.2% of the differences in the data and the two groups of the triplicate pre-contact and post-contact samples clustered together, respectively, albeit with pre-contact replicate 2 being quite removed from pre-contact replicate 1 and replicate 3.

**Figure 7:**
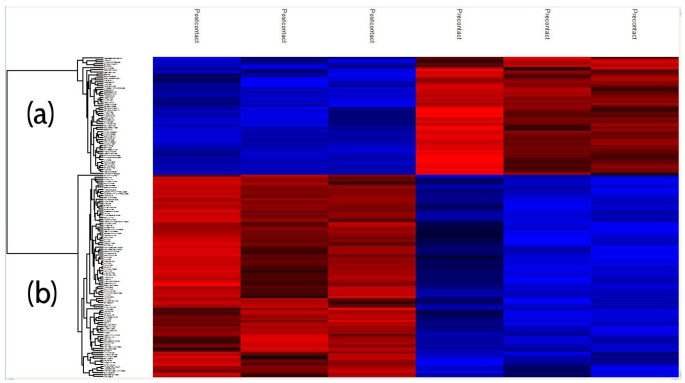
Heat map of the 161 significant peptides identified across all replicates in the pre- and post-contact samples. The significant peptides are clustered into two groups on the y axis; cluster (**a**) is 59 peptides and cluster (**b**) is 102 peptides.

A *t*-test was carried out on the log transformed ion current intensities and for visualization purposes it was normalized. The *t*-test comparison between pre- and post- contact solutions, with a *p*-value of 0.01, of the peptides that were observed in all replicates in both groups yielded 161 peptides significantly different based on the peptide intensity profile. The results of this are visualized in the heat map in Figure 7. The pattern was again observed in which the pre-contact and post-contact clustered together, respectively, with the pre-contact replicate 2 displaying a slightly different profile from the other two replicates. Of these significant peptides, there was a cluster of 102 peptides identified that were relatively enriched in the solution post-contact with the beads (Figure 7b), while 59 identified that were relatively depleted (Figure 7a). 

**Figure 8:**
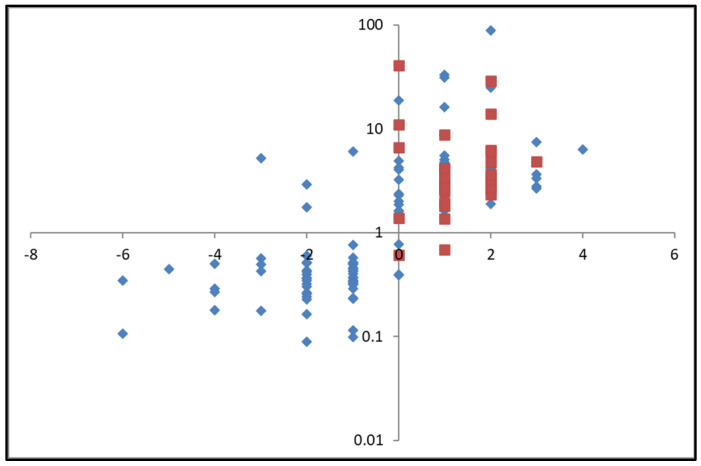
Selective enrichment/depletion of 161 identified significant peptides (◆) and 33 predicted antimicrobial peptides (■) as a function of the peptide structural charge. Post/pre is average intensity on a triplicate ratio.

Focusing on the 161 significant peptides, charge values were assigned to each peptide based on their amino acid composition (−1 for aspartic acid and glutamic acid; +1 for histidine, lysine, arginine). Subsequently, the ratio of the intensity of each peptide post-contact with the beads to that in the original pre-contact solution was calculated and plotted against the charge values (Figure 8). It can be seen in this figure that there was an overall depletion (ratio below 1 on the *y*-axis) for peptides that carried a negative charge. In contrast, there was a relative intensity increase in the post/pre ratio (above 1 on the *y*-axis) for peptides that carried a positive charge, i.e., these peptides were relatively enriched post-fractionation. The commercial copper loaded column used by Megías et al. [27,28] was reported to extract histidine containing peptides from the hydrolysate solutions. Histidine residues are cationic and if these peptides are sought, the current system may be more advantageous than the copper loaded commercial columns used. This is because the multiple wash steps with a final acid rinse to extract the peptides from the copper-loaded column would not be necessary; instead the peptides of interest would be contained in the post-contact solution. Therefore, the chitosan copper bead system may offer a much simpler, clean fractionation system, with reduced processing steps.

The current results corroborate the results for the amino acids and model proteins; the hydrogel bead system seems capable of fractionating based on chemical structure, leaving a post-contact solution that is enriched in cationic amino acids and peptides. Building on this, the previous SEC HPLC results showed the peptides remaining in solution to be proportionally enriched in smaller and medium-sized peptides. Overall, the peptides enriched in the post-contact solution are small- and medium-sized cationic peptides. The bioactivity of peptides is based on their amino acid sequence and structure, and sizes of active sequences are reported to typically range from two to twenty amino acids [29]. Interestingly, small cationic peptides, such as those enriched in the current study, are widely reported to possess biological activities, including behaving as cell penetrating peptides, in addition to possessing antimicrobial activities [25,26]. In fact, when looking at the selective enrichment of the 33 peptides predicted to have antimicrobial properties according to the bioinformatics tools developed by Veltri et al. [30], all but two are shown to be enriched post-contact with the copper chitosan beads (Figure 8) highlighting the potential of these beads to enrich bioactive peptides from a crude hydrolysate.

## 3. Conclusions

The present work introduced copper-crosslinked chitosan gel beads as a simple method for separating amino acids based on chemical interactions. The gel beads exhibited an affinity for amino acids that was both concentration dependent and structure dependent. The extent of immobilization was found to be independent of salt concentration and over a broad range of pH values. The chitosan copper beads were also found to be capable of interacting with intact proteins and a complex hydrolysate. The quantitative results for the hydrolysate fractionation showed good immobilization levels while the qualitative results showed a high affinity for the larger, less hydrolyzed peptides in the hydrolysate. This resulted in post-contact solutions that were relatively enriched in smaller peptides. Further characterization of the small peptides on a sequence level by ESI LCMS showed that the enrichment was systematically biased towards cationic peptides. On combining all of these results it can be concluded that the post-contact solution was enriched extensively in smaller, cationic peptides, characteristics that are widely reported to have cell-penetrating and anti-microbial properties. Bioinformatics tools showed that of the 161 significant peptides present in the hydrolysate, 33 were predicted to possess antimicrobial properties and of these, all but 2 were shown to have been significantly enriched in the post-contact solution.

Overall, the chitosan copper beads present a simple affinity system capable of fractionating complex hydrolysates, with great potential to enhance the content in bioactive peptides.

## 4. Materials and Methods

### 4.1. Materials

Chito-clear FG95, a food grade chitosan manufactured from crustaceans by Primex (Siglufjordur, Iceland) was used (degree of deacetylation 95%, viscosity of 1% *w*/*v* solution in 1% *w*/*v* acetic acid 49 mPa s according to manufacturers, ashes 0.3%). HPLC grade acetonitrile and methanol, and analytical grade sodium hydroxide, glacial acetic acid, riboflavin, sodium chloride (NaCl), sodium tetraborate, O-Phthaldialdehyde reagent incomplete solution (OPA), 2-mercaptoethanol, ≥99.0% (MeSH), amino acids (aspartic acid, histidine, alanine, tryptophan and lysine) and protein standards (β-casein, β-lactoglobulin, α-lactalbumin and insulin β chain) were purchased from Sigma Aldrich (Arklow, Ireland). Sodium caseinate hydrolysate was prepared as previously described [31] using sodium caseinate from Armor Proteins (Loudeac, France).

Sodium phosphate monobasic, sodium phosphate dibasic and cupric sulphate pentahydrate were purchased from Sigma Aldrich (Arklow, Ireland). Deionized water was used for all experiments except for HPLC work when ultrapure water (Millipore, Burlington, MA, USA) was used.

### 4.2. Preparation of Copper-Crosslinked Chitosan Gel Microbeads

The copper-crosslinked chitosan beads were manufactured as described previously [21]. Briefly, a 3% *w*/*v* chitosan solution was prepared in 0.6% *w*/*v* acetic acid solution under magnetic stirring at 500 rpm and left to hydrate overnight at 4 °C. The gel solution was dispensed dropwise through a 100 μL pipette tip using a variable speed transfer pump (Gilson minipuls 2, Villiers-le-Bel, France) at a rate of 0.6 mL per minute into 0.06 M copper sulphate gelling bath to produce beads, circa 2 mm in diameter. The beads were then transferred to a secondary alkaline gelling bath, in which the pH was adjusted to pH 7. The beads were extensively rinsed with deionized water before use. 

### 4.3. Immobilisation of Amino Acids in Gel Beads

The uptake of a range of single amino acids (tryptophan, aspartic acid, histidine, alanine or lysine) into preformed beads was investigated by a series of experiments similar to those described by Egan et al. [22] and O’Neill et al. [23]. Briefly, the beads were left in contact with the amino acid loading solutions (100 µg/mL) for 24 h at room temperature to ensure equilibrium had been reached. After absorption, the supernatant solution was separated by simple filtration of the beads without recourse to centrifugation. The amino acid solutions were analyzed pre- and post-contact with the beads to determine the extent of amino acid immobilization into the beads. Experiments were conducted in which the concentration of chitosan copper (CsCu) polymer was increased over a range of 0 to 40.12 mg/mL dry basis, by increasing the concentration of beads up to 1 g/mL contact solution. As the experiment consists of dropping fresh aqueous gel beads in a small volume of concentrated bioactive solution according to Equation (1) which takes into consideration the volume of the water contributed by the beads (considering the bead phase as miscible with the aqueous phase, thereby diluting it).

(1) $$Immobilisation\;\left(\%\right)=1-\frac{C_{final}\times {(V}_{H_{2}O}+V_{b})}{C_{O\;}\times V_{H_{2}O}}\;\times 100$$

where *V*_*H*_2_*O*_ is the volume of the precontact solution (aqueous phase) and *V_b_* is the volume of the beads, *C_O_* and *C_final_* are the concentrations of amino acids in solution before contact and after 24 h contact, respectively.

The effect of pH on the extent of immobilization into the chitosan copper beads was investigated by working at a fixed chitosan concentration of 3.68 mg/mL dry basis, and adjusting the pH from 2 to 12 by adding small aliquots of HCl or NaOH (0.1 M) solutions.

The effect of salt concentration on the on the extent of immobilization was investigated by a set of experiments in which a fixed amount of gel beads (4 mg/mL dry basis) were added to tryptophan solutions containing a range of NaCl concentrations (0–200 mM).

Amino acid solutions were analyzed with an Agilent 1200 series quaternary HPLC with a fluorescence detector (Agilent Technologies, Santa Clara, CA, USA). Using a LUNA-C18 wide pore column (150 mm × 4.6 mm i.d.; 5 µm particle size from Phenomenex (Cheshire, UK) fitted with a C18 guard column. The mobile phase consisted of acetonitrile and 25 mM phosphate buffer, pH 6.9, controlled at 1 mL/min, where 5% acetonitrile was increased linearly to 60% acetonitrile in 15 min. For simultaneous determination of all amino acids, an online precolumn derivatization injector program was used with the fluoraldehyde OPA reagent. Peaks were detected at an excitation wavelength of 338 nm and emission wavelength of 450 nm.

### 4.4. Immobilisation of Sodium Caseinate Proteins and Hydrolysate

Solutions were prepared by dissolving either 50 mg of hydrolysate or 200 mg Sodium Caseinate (Armor Proteins, France) in 1 mL of ultrapure water. The powder was allowed to dissolve for 2 h at ambient temperature under constant agitation on a magnetic stirrer hotplate. Experiments were conducted in which the concentration of chitosan copper polymer (in the form of fresh beads) was increased over a range of 0 to 40 mg/mL. The beads were left in contact with the solution for 24 h at room temperature to ensure equilibrium had been reached. The concentration of the hydrolysate solution was measured pre- and post-contact with the beads to calculate the extent of uptake by difference, using Equation (1). Protein concentration of the hydrolysate solutions was determined using two methods; firstly, using a UV Vis spectrophotometer (Mason Technology, Dublin, Ireland, Shimadzu, UV mini 1240) at 288 nm using derivative spectroscopy as outlined by Yuan et al. [32]. 

The SEC characterization of the hydrolysate was performed on OPA derivatized sample as per Section 4.3, using a Yarra 3 µm SEC-2000 (300 mm × 7.8 mm) gel filtration chromatography analytical column (Phenomenex, Cheshire, UK). Chromatography was performed with a 20 min isocratic elution at 30 °C and a flowrate of 0.8 mL/min. Peaks were detected at an excitation wavelength of 338 nm and emission at 450 nm. The mobile phase was 45% ACN, 0.1% TFA (*w*/*w*) in ultrapure water. A calibration curve for molecular weight based on retention time was constructed for peptides within the range of 204–24,000 Da. The standards used were β-casein (24,000 Da), β-lactoglobulin (18,400 Da), α-lactalbumin (14,175 Da), insulin β chain (oxidized) (3496 Da) and tryptophan (204 Da). Comparison with the retention times of the standards allowed determination of estimated molecular size distributions of the samples.

### LCMS Analysis of the Post-Contact Solution

Peptide hydrolysates (50 μg) were acidified by trifluoroacetic acid (TFA), desalted with C18 STAGE tips [33], and resuspended in 0.1% TFA.

Peptide fractions were separated using in-house packed reversed-phase C18 columns (C18-AQ Dr. Maisch Reprosil-Pur 100 × 0.075 mm × 3 μm) on a NanoLC Ultimate 3000 HPLC (Dionex LC Packings, now Thermo Scientific, Waltham, MA, USA) and eluted with a linear gradient from 1% to 27% over 60 min at a flow rate of 250 nL/min. This was connected to a quadrupole Orbitrap (Q-Exactive, Thermo Scientific) mass spectrometer operating in data dependent mode. Survey scans (*m*/*z* 300–2000) were acquired in the Orbitrap followed by twelve most intense ions being sequentially isolated and fragmented in MS2 by higher-energy C-trap dissociation. 

Raw data was searched using database matching engine Maxquant (version 1.5.5.1) [34,35] against a custom database of bovine proteins previously identified in the milk proteome containing 1059 entries. Default settings were used, except for unspecific enzyme, no fixed modification and match between runs [36]. 

The Perseus statistical software (version 1.5.5.3) [37] was used to analyze the peptide quantitative values. The data was log transformed and a *t*-test comparison of pre- and post-contact fractions was carried out. Heat maps and principal component analysis (PCA) were employed for visualization of global peptide signatures across the two groups and to examine systematic trends with the data. For the generation of these visualizations, missing values were imputed with values from a normal distribution and the dataset was normalized by z-score.

### 4.5. Data Analysis

All experiments were performed in triplicate. The data obtained were expressed as mean ± S.D. 

## Data Availability

All data and materials are available on request from the corresponding author. The data are not publicly available due to ongoing research using a part of the data.
